# Mass severe acute respiratory coronavirus 2 (SARS-CoV-2) testing of asymptomatic healthcare personnel – ERRATUM

**DOI:** 10.1017/ice.2021.107

**Published:** 2022-09

**Authors:** Scott C. Roberts, David R. Peaper, Craig D. Thorne, L. Scott Sussman, Thomas S. Murray, Steven J. Choi, Christian M. Pettker, Mark B. Russi, Richard A. Martinello

In the above article^1^, the title should read “Mass severe acute respiratory coronavirus 2 (SARS-CoV-2) testing of asymptomatic healthcare personnel”
